# Suitability of Polymyxin B as a Mucosal Adjuvant for Intranasal Influenza and COVID-19 Vaccines

**DOI:** 10.3390/vaccines11111727

**Published:** 2023-11-18

**Authors:** Naoto Yoshino, Takuya Yokoyama, Hironori Sakai, Ikumi Sugiyama, Takashi Odagiri, Masahiro Kimura, Wataru Hojo, Tomoyuki Saino, Yasushi Muraki

**Affiliations:** 1 Division of Infectious Diseases and Immunology, Department of Microbiology, School of Medicine, Iwate Medical University, 1-1-1 Idaidori, Yahaba 028-3694, Iwate, Japan; 2 Department of Anatomy (Cell Biology), Iwate Medical University, 1-1-1 Idaidori, Yahaba 028-3694, Iwate, Japan; 3 Laboratory of Veterinary Anatomy and Cell Biology, Faculty of Agriculture, Iwate University, 3-18-8 Ueda, Morioka 020-8550, Iwate, Japan; 4 R&D, Cellspect Co., Ltd., 2-4-23 Kitaiioka, Morioka 020-0857, Iwate, Japan; 5 Division of Advanced Pharmaceutics, Department of Clinical Pharmaceutical Science, School of Pharmacy, Iwate Medical University, 1-1-1 Idaidori, Yahaba 028-3694, Iwate, Japan

**Keywords:** polymyxin B, mucosal adjuvant, hemagglutinin, S1 subunit, S protein, influenza A virus, SARS-CoV-2

## Abstract

Polymyxin B (PMB) is an antibiotic that exhibits mucosal adjuvanticity for ovalbumin (OVA), which enhances the immune response in the mucosal compartments of mice. Frequent breakthrough infections of severe acute respiratory syndrome coronavirus 2 (SARS-CoV-2) variants indicate that the IgA antibody levels elicited by the mRNA vaccines in the mucosal tissues were insufficient for the prophylaxis of this infection. It remains unknown whether PMB exhibits mucosal adjuvanticity for antigens other than OVA. This study investigated the adjuvanticity of PMB for the virus proteins, hemagglutinin (HA) of influenza A virus, and the S1 subunit and S protein of SARS-CoV-2. BALB/c mice immunized either intranasally or subcutaneously with these antigens alone or in combination with PMB were examined, and the antigen-specific antibodies were quantified. PMB substantially increased the production of antigen-specific IgA antibodies in mucosal secretions and IgG antibodies in plasma, indicating its adjuvanticity for both HA and S proteins. This study also revealed that the PMB-virus antigen complex diameter is crucial for the induction of mucosal immunity. No detrimental effects were observed on the nasal mucosa or olfactory bulb. These findings highlight the potential of PMB as a safe candidate for intranasal vaccination to induce mucosal IgA antibodies for prophylaxis against mucosally transmitted infections.

## 1. Introduction

Polymyxin B (PMB) is a clinically used antibiotic. Using a drug-repositioning strategy [1], we observed that PMB elicits mucosal adjuvanticity; PMB evoked much higher titers of ovalbumin (OVA)-specific IgA antibodies (Abs) in the mucosal compartments of intranasally immunized mice [2]. We also elucidated the mechanism underlying the adjuvanticity [2]: (1) the diameter of the PMB-OVA complex formed in the immunization solution is suitable for the induction of mucosal immunity, and (2) PMB induces mast cell degranulation, leading to the activation of innate immunity and enhanced acquired immunity. However, the adjuvanticity of PMB for virus proteins remains unknown.

Coronavirus disease 2019 (COVID-19) caused by the novel severe acute respiratory syndrome coronavirus 2 (SARS-CoV-2) emerged in late 2019 and was declared a pandemic in March 2020. It has since become a major public health concern [3,4], with 6.7 million deaths (as of 10 March 2023) [5]. The intramuscular administration of COVID-19 vaccines is reported to be effective, as fully vaccinated individuals experience decreased hospitalization and mortality compared to non-vaccinated individuals with similar risk factors [6,7,8,9]. However, breakthrough infections in vaccinated individuals have been frequently reported [10,11,12,13]. Before the COVID-19 pandemic, annual epidemics of influenza occurred worldwide, primarily in winter, despite the global use of intramuscular or subcutaneous influenza vaccines (whole inactivated virus or hemagglutinin [HA] split) [14,15,16]. This suggests that the plasma IgG Abs induced by these vaccines do not necessarily protect against virus infections.

A crucial step in controlling mucosally transmitted infections, such as COVID-19 and influenza, is achieving immunoprophylaxis in mucosal tissues via mucosal immunization. This induces pathogen-specific immunoprophylaxis primarily by inducing the production of secretory IgA Abs in the mucosal tissues [17,18]. Particularly, intranasal vaccination is highly potent at inducing antigen-specific IgA Abs in the respiratory tract [19]. Furthermore, mucosal adjuvants for subunits or recombinant protein antigens are required to induce a potent immune response; however, no mucosal adjuvants are currently available for clinical use [20].

The present study investigated whether PMB has adjuvanticity for antigens other than OVA. We used BALB/c mice to determine the suitability of PMB as a mucosal adjuvant for the following virus proteins: influenza A virus hemagglutinin (HA) and the S1 subunit and S protein of SARS-CoV-2. Furthermore, we analyzed the diameter of the PMB-virus antigen complex, which is a representative physicochemical property for efficient mucosal adjuvanticity [21]. Additionally, we assessed the histology of the nasal cavity, including that of the olfactory bulb, in intranasally immunized mice.

## 2. Materials and Methods

### 2.1. Ethics Statement

All animal experiments in the present study were approved by the Committee on the Ethics of Animal Experiments (CEAE) of the Iwate Medical University (Permit No. 02-013). The animal experiments were performed in compliance with the recommendations of the Guidelines for Proper Conduct of Animal Experiments established by the Science Council of Japan and the regulations established by the CEAE.

### 2.2. Antigens and Adjuvant

The HA split of mouse-adapted (MA)-influenza A/Iwate/1130/2009 (H1N1pdm09) [22] was generated and provided by the Research Foundation for Microbial Diseases of Osaka University (BIKEN), Kagawa, Japan; the MA-A/Iwate/1130/2009 virus was propagated in MDCK cells, and the culture supernatant was purified via filtration (0.45 μm) followed by sedimentation using a linear sucrose gradient [23]. The recombinant S1 subunit (amino acids 251–660) of the SARS-CoV-2 Wuhan-Hu-1 strain expressed in *Escherichia coli* was purchased from FAPON Biotech Inc. (Donggua, China). The recombinant S protein (amino acid 16–1213) of the SARS-CoV-2 Wuhan-Hu-1 strain expressed in HEK239 cells was purchased from ACROBiosystems (Newark, DE, USA). Polymyxin B sulfate (FUJIFILM Wako Pure Chemical Co., Osaka, Japan) was used as the mucosal adjuvant.

### 2.3. Immunization of Mice

Five-week-old female BALB/cAJcl mice (18–21 g) were purchased from CLEA Japan (Tokyo, Japan). The mice were acclimated to the laboratory animal facility for 1 week prior to the experiments.

The virus antigens and PMB were dissolved in normal saline (<0.25 endotoxin unit/mL; Otsuka Pharmaceutical Factory, Tokushima, Japan). Immunization solutions were prepared using 1 μg of HA split, 1 μg of S1 subunit, 10 μg of S1 subunit, or 1 μg of the S protein with or without 500 μg of PMB in 10 or 50 µL aliquots. Virus antigens were first dissolved in normal saline at the following concentrations: 0.2 mg/mL (HA), 0.2 mg/mL (S1), 2 mg/mL (S1), and 0.2 mg/mL (S). A 100 mg/mL PMB solution in normal saline was then prepared. Equal volumes of the virus antigen solutions (for each of the antigens) and the PMB solution (or normal saline for antigen alone) were mixed to prepare samples for intranasal (IN) immunization. For subcutaneous (SC) immunization, the nasal immunization samples were diluted 5-fold with normal saline. All immunization samples were prepared at the time of use.

The mice were mildly anesthetized with ketamine (Ketarar^®^; Daiichi Sankyo Co., Ltd., Tokyo, Japan) and randomly divided into IN or SC immunization groups. For IN immunization, mice were administered a 10 µL aliquot (5 µL/nostril) of the immunization solution, whereas SC immunization was performed by injecting a 50 µL aliquot of the immunization solution into the interscapular region of the mice. Each mouse was immunized thrice at 0, 7, and 14 days, as previously described [2,21,24].

A week after the last immunization, mucosal secretions (tracheal–bronchial lavage [TBL], nasal washes [NWs], saliva, fecal extracts [FEs]), and plasma samples were collected, as previously described [25,26]. Vaginal washes (VWs) were collected as per the method reported by Wu et al. [27], with minor modifications. Briefly, 50 μL saline was injected into the vagina of the mice and withdrawn using a 200 μL pipettor. The procedure was repeated four times to obtain a total volume of approximately 200 μL of VW sample. The mice were euthanized directly via cervical dislocation under anesthesia with 3.0% isoflurane and 0.5 L/min oxygen.

### 2.4. Enzyme-Linked Immunosorbent Assay (ELISA)

The anti-HA, anti-S1, and anti-S Abs in the samples were quantified using ELISA. The anti-HA and anti-S Ab measurements were performed by coating the plates with the HA and S proteins used for immunization. The experiments were performed as previously described [28]. For quantifying the anti-S1 Ab, we employed the COVID-19 Human IgM IgG ELISA Kit (Spike Protein) (R&D Cellspect Co., Ltd., Morioka, Japan) and coated the wells with five times the amount of antigen (S1 subunit) used for anti-HA and anti-S Ab measurements; the subsequent procedure followed was the same as that described previously [28].

Mouse anti-HA monoclonal Ab (mAb) against influenza A/California/06/2009 (H1N1pdm09) (eEnzyme LLC., Gaithersburg, MD, USA) and mouse anti-S1 (Wuhan-Hu-1; SARS-CoV-2 spike neutralizing Ab) IgG mAb (Sino Biological, Inc., Beijing, China) were used to determine the detection limit of the ELISA systems. The detection limits for the anti-HA, anti-S1, and anti-S Abs were found to be 6.25 ng/mL, 6.25 ng/mL, and 0.3125 ng/mL, respectively. Additionally, no nonspecific reactions were observed in the ELISA systems using naïve BALB/c mouse plasma diluted 1:2^6^ (×64) and NWs diluted 1:2 (×2).

### 2.5. Particle Diameter

The particle diameters of the virus antigen and PMB-virus antigen complex were measured using a Zetasizer Nano ZS system (Malvern Instruments Ltd., Worcestershire, UK), as described previously [21,24].

### 2.6. Histopathological Analysis

A week after the last immunization, the head of each mouse was collected and fixed in 10% neutral-buffered formalin. After washing with PBS, the specimens were decalcified in 10% ethylenediamine-N,N,N’,N’-tetraacetic acid disodium (pH 7.0) for 1 week at 4 °C. After washing with PBS, the frontal sections were cut into 5 mm thick slices and embedded in paraffin. Thereafter, 10 μm thick histological sections were stained with Mayer Hematoxylin for 12 min and Eosin for 2 min. The slides were scanned using a virtual slide scanner (NanoZoomer^®^-RS; Hamamatsu Photonics, Hamamatsu, Japan) at 40× magnification and visualized using a viewer software (Hamamatsu Photonics). The thickness and morphological changes of each layer in the nasal mucosal tissue and olfactory bulb, as well as the infiltration of leukocytes into the tissue, were analyzed. The safety of IN immunization was evaluated compared to the SC immunization group.

### 2.7. Statistical Analysis

A one-way analysis of variance (ANOVA) was used to compare the three groups. If the one-way ANOVA identified a significant effect in a group, a post hoc Tukey’s multiple comparison test was performed. Differences between groups were considered significant at a *p*-value of <0.05. Statistical analyses were performed with GraphPad Prism 9.5.1 (528) (GraphPad Software, Inc., Boston, MA, USA).

## 3. Results and Discussion

### 3.1. Immunization of Mice with Influenza HA Split

In the present study, since influenza and COVID-19 vaccines are injectable vaccines, subcutaneous (SC) inoculation was used as a control for comparison with the intranasal (IN) vaccine. In addition, the major adverse effects of injected PMB are severe nephrotoxicity and neurotoxicity. Owing to the apparent toxicity, SC inoculation with PMB was not performed.

**Figure 1:**
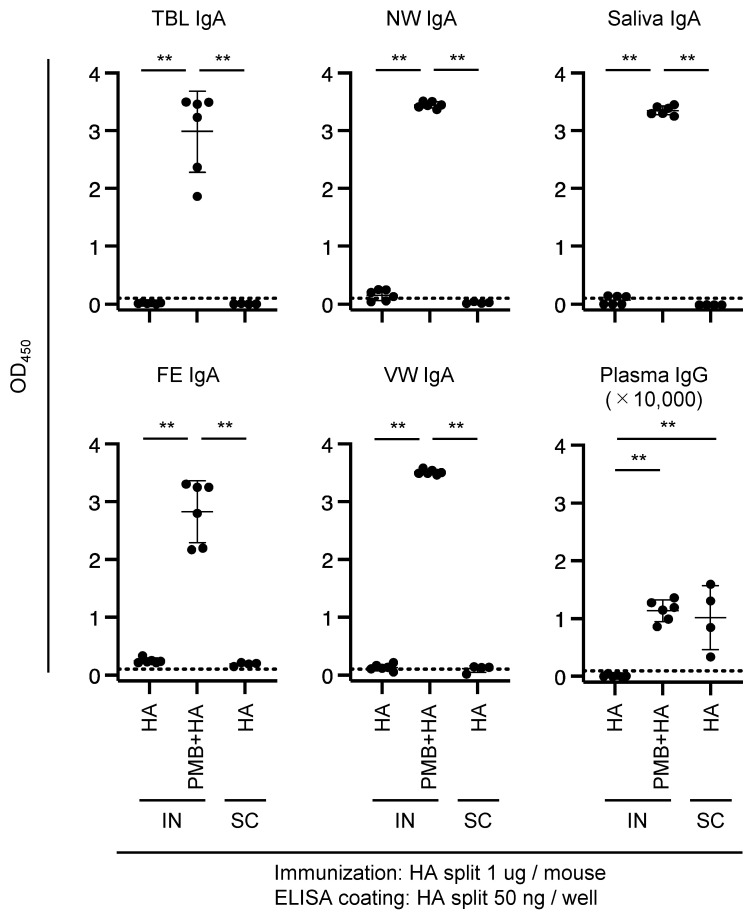
Quantity of HA-specific Ab in mice immunized with HA split. Mice were subjected to intranasal (IN) or subcutaneous (SC) immunization with 1 μg of HA split (4–6 mice per experimental group). Mucosal secretions (TBL: tracheal–bronchial lavages; NW: nasal washes; saliva; FE: fecal extract; VW: vaginal wash) and plasma were collected. HA-specific IgA and IgG Abs in mucosal secretions (1:2) or plasma (1:10,000) diluted in 1% BSA-PBS were quantified using an ELISA system described in the Materials and Methods section. Each dot represents a single mouse. The horizontal dotted line indicates the detection limit. Significant differences in OVA-specific Ab titers between experimental groups are indicated by asterisks: ** *p* < 0.005.

Female BALB/c mice were administered influenza HA split intranasally or subcutaneously, and the Abs were quantified using an ELISA system. The quantity of HA-specific IgA Abs in the HA (IN) and HA (SC) group mice was at or below the detection limit (Figure 1). In contrast, IN administration of PMB + HA resulted in a significant increase in the IgA levels in the TBL, NW, saliva, FE, and VW samples. Furthermore, plasma IgG levels increased significantly in the PMB + HA (IN) and HA (SC) groups compared with those in the HA (IN) group. However, no significant difference was observed in the amount of plasma IgG Abs between PMB + HA (IN) and HA (SC) groups, indicating that PMB administered intranasally with HA elicited systemic immunity to the same extent as HA administered subcutaneously.

**Figure 2:**
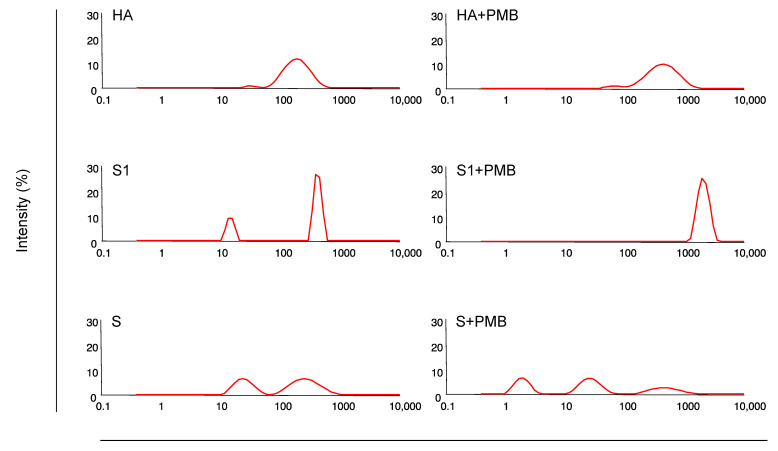
Particle diameter of PMB-virus antigen complex. Influenza HA split (1 μg), SARS-CoV-2 S1 subunit (1 μg), or SARS-CoV-2 S protein (1 μg) was mixed with PMB (500 μg) in saline. Particle diameters of the complexes were measured using a Zetasizer Nano ZS system. X- and y-axes indicate particle diameter and intensity, respectively.

We previously reported that the particle diameter of the OVA-PMB complex is related to the induction level of mucosal immunity [21]. Therefore, we measured the particle diameters of HA split with or without PMB in the immunization solutions (Figure 2, Table 1). The diameter of the distribution with the largest proportion of particles (dominant diameter) in the HA and PMB + HA groups was 202.4 and 418.0 nm, respectively. This significant difference in the dominant diameters between the HA and PMB + HA groups (*p* < 0.0005, unpaired Student’s *t*-test) indicates that PMB formed a complex with HA in the solution. Furthermore, the dominant diameter in the PMB + HA group (418 nm) was within the appropriate range for eliciting mucosal immune responses (100–500 nm) [29]. Thus, we concluded that PMB efficiently induces mucosal immunity against HA by forming a complex with the antigen protein.

Mast cells are required for efficient mucosal immunity [30]; their role in eliciting mucosal immunity should be evaluated. We previously showed that surfactin (lipopeptide analog of PMB) elicits a higher level of IgA Ab production by activating mast cells in the nasal mucosa [21]. Although the dominant diameter of HA (202.4 nm) (Figure 2, Table 1) was appropriate for translocation into the nasal mucosa, the IgA Abs in mice immunized with HA (IN) was at or below the detection limit (Figure 1). This may be attributed to the absence of mast cell activation by PMB, resulting in the inefficient activation of innate immunity and, subsequently, acquired immunity.

### 3.2. Immunization of Mice with SARS-CoV-2 S1 Subunit

Our findings indicate that PMB elicits mucosal adjuvanticity for HA split (Figure 1), prompting us to examine whether PMB elicits adjuvanticity for the SARS-CoV-2 S protein. Initially, we used the S1 subunit as an immunization antigen because S1 contains the receptor-binding domain necessary for binding to the human ACE2 receptor and subsequent virus entry; it, therefore, contains most epitopes targeted by neutralizing Abs [31,32]. In the present study, the S1 subunit (1 µg) was administered with or without PMB (500 µg) to mice intranasally or subcutaneously, and the IgA and IgG Abs were quantified (Figure S1). The IgA Ab level was below the detection limit in all the examined mice, whereas IgG Abs could be detected in the plasma of mice immunized subcutaneously.

We assumed that 1 µg of the S1 subunit was insufficient for eliciting mucosal immunity; therefore, we increased the dosage to 10 µg of the S1 subunit and immunized the mice in the same manner. As shown in Figure S2, the amount of IgG Ab in the plasma of mice immunized subcutaneously increased in a dose-dependent manner compared with the previous values (Figure S1) (*p* < 0.005, unpaired Student’s *t*-test). However, the quantity of IgA Abs in the various mucosal secretions was below the detection limit.

The observation that the S1 subunit did not elicit mucosal immunity was confirmed using an ELISA system in which the S protein was used as an antigen. The aliquots mentioned in Figure S2 (from mice immunized with 10 µg of the S1 subunit) were examined. The IgA Ab levels in the TBL and NW were below the detection level (Figure S3). Thus, we concluded that the S1 subunit is immunogenic in mice but unable to elicit a mucosal immune response.

The intrinsic nature of the S1 subunit appears to be involved in eliciting inefficient mucosal immunity (Figures S1–S3). Given that the length and width of the trimeric S protein are 21 nm and 8.7 nm, respectively [33], the diameter of the S1 subunit was likely smaller. Nevertheless, the dominant diameter of the S1 subunit and PMB + S1 in the immunization solution was 391.4 nm and 2209.3 nm, respectively (Figure 2, Table 1), suggesting that the S1 subunit forms aggregates and that these S1 aggregates form complexes with PMB to form particles with large diameters. In the present study, we used *E. coli* derived-S1 with no glycans attached. Based on a previous report that glycans on bovine serum albumin molecules suppress self-aggregation [34], we speculate that the S1 subunit is readily aggregated owing to the lack of glycans, which leads to the formation of larger particles, resulting in inefficient mucosal immunity.

### 3.3. Immunization of Mice with SARS-CoV-2 S Protein

The results shown in Figures S1–S3 indicate that the S1 subunit is not a viable candidate for IN immunization in mice. Therefore, we immunized the mice with the S protein (1 µg) with or without PMB, and the IgA and IgG Abs induced against the S1 subunit were quantified. Immunization in the PMB + S (IN) group resulted in a significant increase in IgA Ab levels in the VW and IgG Ab levels in the plasma compared with that in the S (IN) group (*p* < 0.005); however, the corresponding OD_450_ values were <1.0 (Figure S4).

**Figure 3:**
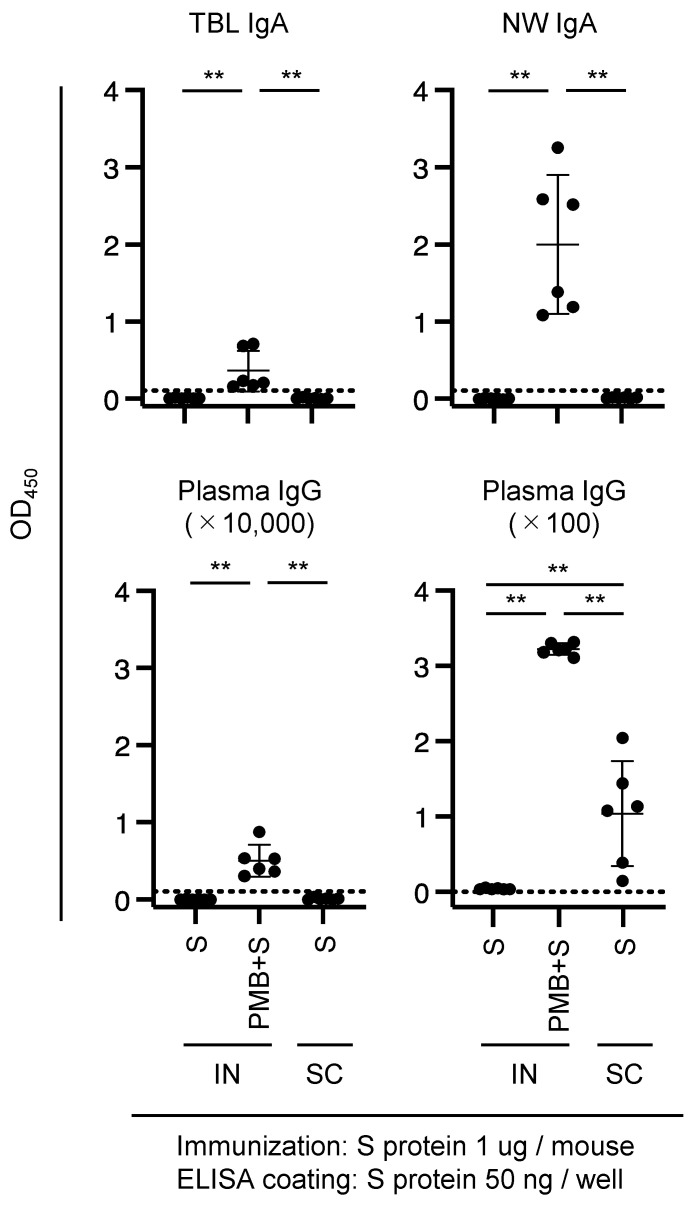
Amount of S-specific Ab in mice immunized with 1 μg of S protein. Mice were subjected to intranasal (IN) or subcutaneous (SC) immunization with 1 μg of the SARS-CoV-2 S protein (4–6 mice per experimental group). The S-specific Abs in the mucosal secretions (1:2) or plasma (1:10,000 or 1:100) diluted in 1% BSA-PBS were quantified using ELISA. ** *p* < 0.005.

The low OD values shown in Figure S4 can be attributed to using different antigens (S protein for immunization and S1 subunit for ELISA quantification). Therefore, we quantified the Abs against the S protein in the ELISA system. An aliquot of each of the TBL, NW, and plasma samples (Figure S4) was used to quantify the specific anti-S Abs using this system. We detected an increased amount of S-specific IgA Abs in TBL and NW samples from the mice in the PMB + S (IN) immunization group compared with those in the S (IN) group (Figure 3, upper panels), indicating that PMB possesses mucosal adjuvanticity for the S protein.

Although the quantity of S-specific plasma IgG Abs in the 10,000-fold diluted plasma sample was at or below the detection limit (Figure 3, lower left panel), IgG Abs pertaining to the PMB + S (IN) and S (SC) groups were unequivocally detected in the 100-fold diluted plasma samples (Figure 3, lower right panel). Furthermore, similar to the level of anti-HA plasma IgG Abs (Figure 1, lower right panel), a substantial level of plasma IgG Abs was detected in mice in the PMB + S (IN) group (Figure 3, lower right panel). Thus, IN immunization with PMB elicits systemic immunity against the S protein and mucosal immunity.

The distribution of diameters for the PMB + S complex was trimodal (Figure 2, Table 1). In the PMB + S solution, some particles exhibited a peak diameter of 470.4 nm, which is within the appropriate range, although this proportion (17.0%) was not predominant. The lower proportion of PMB + S (17.0%) compared with that of PMB + HA (97.7%) may reflect the difference in the OD values of TBL and NW IgA Abs between PMB + S (Figure 3) and PMB + HA (Figure 1).

### 3.4. Histopathology of the Nasal Mucosa of Immunized Mice

**Figure 4:**
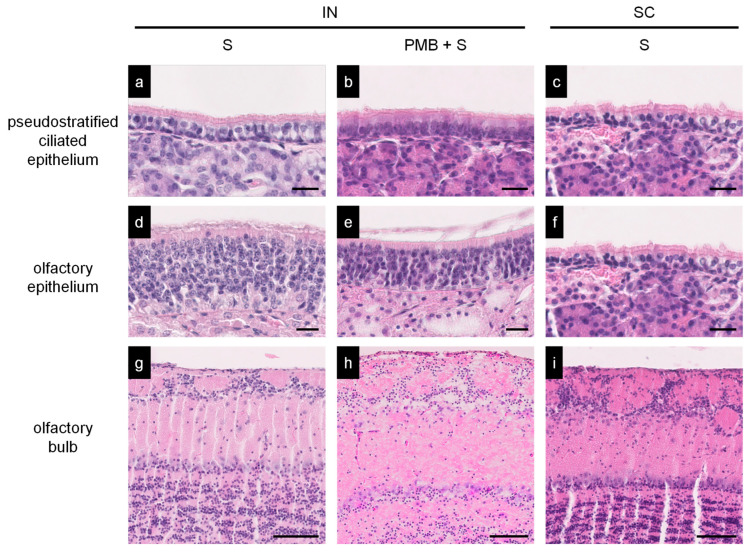
Histopathology of the nasal mucosa of immunized mice. Nasal mucosa (pseudostratified ciliated epithelium and olfactory epithelium) and olfactory bulbs of immunized mice in the S (IN) (**a**,**d**,**g**), PMB + S (IN) (**b**,**e**,**h**), or S (SC) (**c**,**f**,**i**) groups were examined using hematoxylin-eosin (HE) staining. Bar; 20 μm.

We examined the histopathology of the immunized mice to assess the inflammatory responses in the nasal mucosa (pseudostratified ciliated epithelium and olfactory epithelium) and olfactory bulb. The mice were euthanized on day 7 after the last immunization, and the samples were collected. We observed no apparent inflammatory lesions in the intranasal regions where the mice were immunized with PMB + S1 or PMB + S. Similarly, no pathological changes were detected in the olfactory bulb (Figure 4). Therefore, we concluded that PMB + S or S did not exert detrimental effects on the nasal membrane and olfactory bulb, at least with respect to the immunization protocol used in the present study.

The safe attainment of a sufficient IgA Ab concentration on the mucosal membrane is important for the prophylaxis of mucosally transmitted infections. Recently, mucosal IgA Abs associated with intramuscular mRNA vaccination has been detected [35,36,37]. However, the frequent breakthrough infections of variants [10,11,12,13] indicate that the IgA Ab levels in mucosal tissues elicited by the mRNA vaccines were insufficient for prophylaxis of the infection. Thus, IN immunization is required to achieve sufficient levels of IgA Abs in the nasal mucosa. The results of the present study show that PMB is a promising mucosal adjuvant candidate for IN vaccination with an inactivated virus component.

Safety concerns have also necessitated the exploration of alternative options for IN immunization. Bacterial toxins such as cholera toxin (CT) elicit potent mucosal adjuvanticity [38]. It has been reported that the mucosal adjuvanticity of CT was approximately 1.5 times higher than that of PMB [28]. However, mice immunized intranasally with antigens and CT showed severe inflammatory responses in the subepithelial nasal mucosal tissue [28,39]. Consequently, CT is not currently used in clinical settings. In another instance, a heat-labile toxin from *E. coli* was approved as an influenza nasal vaccine for humans but was withdrawn owing to severe side effects such as facial palsy [40]. A live-attenuated influenza vaccine (FluMist^®^) has been approved [41] and is currently available in the USA and Europe. However, from a safety standpoint, an age limit has been imposed for this vaccination [42]. In contrast, PMB, which is an inhalational antibiotic, has been shown to be safe with encouraging clinical results in humans [43,44]; furthermore, the present study has confirmed its efficacy as a mucosal adjuvant. The adverse effects of PMB as an adjuvant are considered to be similar to those of PMB in clinical use. Thus, this drug-repositioning strategy presented here may be a new approach for the development of novel mucosal adjuvants.

Previous studies have demonstrated that the mechanisms of PMB as an adjuvant are (1) complex formation with a particle diameter suitable for transfer to mucosal tissues and (2) mast cell activation [21]. More recently, more potent adjuvants have been developed by combining several compounds with immunostimulatory or antigen-delivery properties. PMB has both immunostimulatory and antigen-delivery properties, making it a suitable mucosal adjuvant. The mechanism by which PMB enhanced specific Ab titers is attributed to its adjuvanticity, and PMB alone would not induce a virus-specific acquired immune response. We observed the availability of PMB as a mucosal adjuvant [22]; IN immunization with PMB plus an antigen (inactivated influenza virus) inhibited disease progression and reduced virus titers in the respiratory tract after lethal doses of influenza virus challenge infection, compared to IN immunization with inactivated influenza virus alone. Furthermore, IN administration of PMB alone caused disease progression and death of mice in all cases, as in naïve mice. Although we have not conducted experiments on SARS-CoV-2 infection, we assume the results will be similar to those of influenza.

PMB is an amphiphilic structure consisting of a hydrophilic head of polypeptide and a hydrophobic tail of alkyl chains [2]. The PMB-virus antigen complex is presumed to be formed by the hydrophobic interaction of the hydrophobic tail of the PMB with the virus antigen. The complex has the virus antigen in the center and is surrounded by hydrophobic tails on the virus antigen side and hydrophilic heads on the solvent side. Particles with a 100–500 nm diameter are considered suitable for inducing mucosal immunity in IN immunization [29]. Furthermore, our previous studies using surfactants and OVA suggest that the diameter of the surfactant-OVA complex affects their adjuvanticity [24]. Although we did not confirm the transfer of PMB-virus antigen complexes into mucosal tissues in the present study, previous findings on the relationship between particle diameter and adjuvanticity suggest that the diameter of a suitable PMB-virus antigen complex would affect vaccine efficacy.

Mucosal immunization with PMB induces specific IgA Ab responses in diverse mucosal tissues. Exposure to antigens via the mucosal route leads to the generation of specific IgA responses both locally and at remote mucosal sites [45]. However, as the mucosal immune system exhibits compartmentalization, mucosal immunity is not always induced equally across different mucosal tissues; for example, IN immunization primarily elicits Ab responses in the upper respiratory tract and cervicovaginal mucosa, whereas the gut is less likely to evoke such immune responses [46,47]. Nevertheless, our previous investigation involving mice immunized intranasally with PMB and OVA demonstrated the presence of OVA-specific IgA Abs in not just the NWs and VWs but in the fecal extracts and saliva as well [2]. Similarly, in the present study, mucosal immunization resulted in the detection of specific IgA Abs in mucosal tissues, which is typically challenging to stimulate via IN immunization. These findings suggest that, in addition to its potential effectiveness against respiratory tract infections, IN vaccination using PMB may be effective against gut infections and sexually transmitted diseases.

PMB did not necessarily exhibit adjuvanticity for all three virus proteins; the focus of the present study (influenza HA and SARS-CoV-2 S1 subunit and S protein) may not necessarily apply to all virus proteins. Mixing antigen and PMB results in a particular particle diameter, but why particles of that diameter are formed (the factors that determine the diameter) remains unknown. We measured particle diameters in solutions of OVA mixed with 31 different surfactants but did not identify factors that determine the diameter [24,48]. Thus, the inability to artificially adjust the diameter of the PMB-protein complex is a limitation of PMB. In addition, the present study lacks an investigation into the long-term effects and durability of the immune response induced by PMB as a mucosal adjuvant. Further, the results obtained from the BALB/c mice model may not directly translate to human responses. Additionally, the potential side effects or safety concerns associated with the use of PMB as a mucosal adjuvant need further evaluation.

The present study has two innovations. First, we demonstrated that compounds with mucosal adjuvanticity are present in clinically used and safe drugs. Second, we applied the findings on nasal drug delivery systems to developing mucosal adjuvants. In the present study, we demonstrated that the physicochemical properties (particle diameter) of the antigen-adjuvant complex affect the adjuvant action. This finding will facilitate the prediction of adjuvanticity by measuring particle diameter in vitro, thus contributing to a reduction in the experimental animals. Furthermore, this study highlights the potential application of drug repositioning strategies in adjuvant discovery to develop safe vaccines.

## 4. Conclusions

We demonstrated that PMB significantly increased the production of antigen-specific IgA Abs in the various mucosal secretions of immunized mice, indicating the mucosal adjuvanticity of PMB for influenza HA and SARS-CoV-2 S proteins. Furthermore, we detected a relationship between the dominant diameter of the PMB-virus protein complex and mucosal adjuvanticity. These findings have broadened our previous observations regarding the mucosal adjuvanticity of PMB for OVA and the relationship between the diameter of the PMB-OVA complex and mucosal adjuvanticity. Consequently, PMB may be suitable for use in influenza and COVID-19 intranasal vaccinations.

## Figures and Tables

**Table 1:** Particle diameter distribution of virus antigens with or without polymyxin B (PMB).

Antigen	PMB	Diameter Distribution by Intensity						PDI ^b^
		Peak 1 ^a^		Peak 2		Peak 3		
		D (nm) ^c^	Int (%) ^d^	D (nm)	Int (%)	D (nm)	Int (%)	
HA	–	202.4 ± 2.3	98.9 ± 1.0	39.2 ± 1.8	1.1 ± 1.0	N/A	N/A	0.207 ± 0.005
	+	418.0 ± 16.9	97.7 ± 2.0	69.1 ± 2.5	2.3 ± 2.0			0.315 ± 0.025
S1	–	391.4 ± 13.1	63.4 ± 1.8	16.3 ± 1.8	36.6 ± 1.8	N/A	N/A	0.737 ± 0.081
	+	2209.3 ± 86.7	100.0	N/A	N/A			0.161 ± 0.051
S	–	246.6 ± 4.4	56.7 ± 1.9	24.5 ± 0.7	43.3 ± 1.9	N/A	N/A	0.610 ± 0.132
	+	26.0 ± 1.7	48.6 ± 2.0	2.0 ± 0.0	34.3 ± 6.8	470.4 ± 79.0	17.0 ± 5.6	0.446 ± 0.139

Note. HA: hemagglutinin; S1: S1 subunit; S: S protein; PMB: polymyxin B. ^a^ The values of peak diameter are shown as the mean ± SD of three independent experiments. ^b^ PDI; polydispersity index. ^c^ D; diameter. ^d^ Int; intensity. N/A, not available.

## Data Availability

The corresponding author had full access to all the data in the study.

## Supplementary Materials

The following supporting information can be downloaded at https://www.mdpi.com/article/10.3390/vaccines11111727/s1. Figure S1: Quantity of S1-specific antibodies (Abs) in mice immunized with 1 μg of S1 subunit; Figure S2: Quantity of S1-specific Abs in mice immunized with 10 μg of S1 subunit; Figure S3: Quantity of S-specific Abs in mice immunized with 10 μg of S1 subunit; Figure S4: Quantity of S1-specific Abs in mice immunized with 1 μg of S protein.
