# Supplementary material for: Suitability of Polymyxin B as a Mucosal Adjuvant for Intranasal Influenza and COVID-19 Vaccines
Source: Vaccines (Basel). 2023 Nov 18;11(11):1727. doi: 10.3390/vaccines11111727 (PMC10675063; doi:10.3390/vaccines11111727)
Supplement: Supplementary file 1 [file vaccines-11-01727-s001.zip › Supplementary Figure Captions.pdf]

Suitability of Polymyxin B as a Mucosal Adjuvant for Intranasal Influenza and COVID-19 Vaccines

Supplementary Figure Captions

Figure S1. Quantity of S1-specific antibodies (Abs) in mice immunized with 1 μg of S1 subunit

Mice were subjected to either intranasal (IN) or subcutaneous (SC) immunization with 1 μg of SARS-CoV-2 S1 subunit (4–6 mice per experimental group). Mucosal secretions (TBL: tracheal-bronchial lavages; NW: nasal washes; saliva; FE: fecal extract; VW: vaginal wash) and plasma were collected. S1-specific IgA and IgG Abs in mucosal secretions (1:2) or plasma (1:100) diluted in 1% BSA-PBS were quantified using an ELISA system described in the Materials and Methods section. Each dot represents a single mouse. The horizontal dotted line indicates the detection limit. Significant differences in OVA-specific Ab titers between experimental groups are indicated by asterisks: ***p*<0.005.

Figure S2. Quantity of S1-specific Abs in mice immunized with 10 μg of S1 subunit

Mice were subjected to either intranasal (IN) or subcutaneous (SC) immunization with 10 μg of S1 subunit of the SARS-CoV-2 S protein (4–6 mice per experimental group). S1-specific Abs in the mucosal secretions (1:2) or plasma (1:100) diluted in 1% BSA-PBS were quantified using ELISA. Abbreviations are the same as those in the legend of Figure S1. ***p*<0.005.

Figure S3. Quantity of S-specific Abs in mice immunized with 10 μg of S1 subunit

Aliquots of the samples in Figure S2 were used to quantify the S-specific antibodies using ELISA. Abbreviations are the same as those in the legend of Figure S1. ***p*<0.005.

Figure S4. Quantity of S1-specific Abs in mice immunized with 1 μg of S protein

Mice were subjected to either intranasal (IN) or subcutaneous (SC) immunization with 1 μg of SARS-CoV-2 S protein (4–6 mice per experimental group). S1-specific Abs in mucosal secretions (1:2) or plasma (1:100) diluted in 1% BSA-PBS were quantified using ELISA. Abbreviations are the same as those in the legend of Figure S1. **p*<0.05, ***p*<0.005.
