# Supplementary material for: Suitability of Polymyxin B as a Mucosal Adjuvant for Intranasal Influenza and COVID-19 Vaccines
Source: Vaccines (Basel). 2023 Nov 18;11(11):1727. doi: 10.3390/vaccines11111727 (PMC10675063; doi:10.3390/vaccines11111727)
Supplement: Supplementary file 1 [file vaccines-11-01727-s001.zip › Supplementary Figures.pptx]

## Slide 1
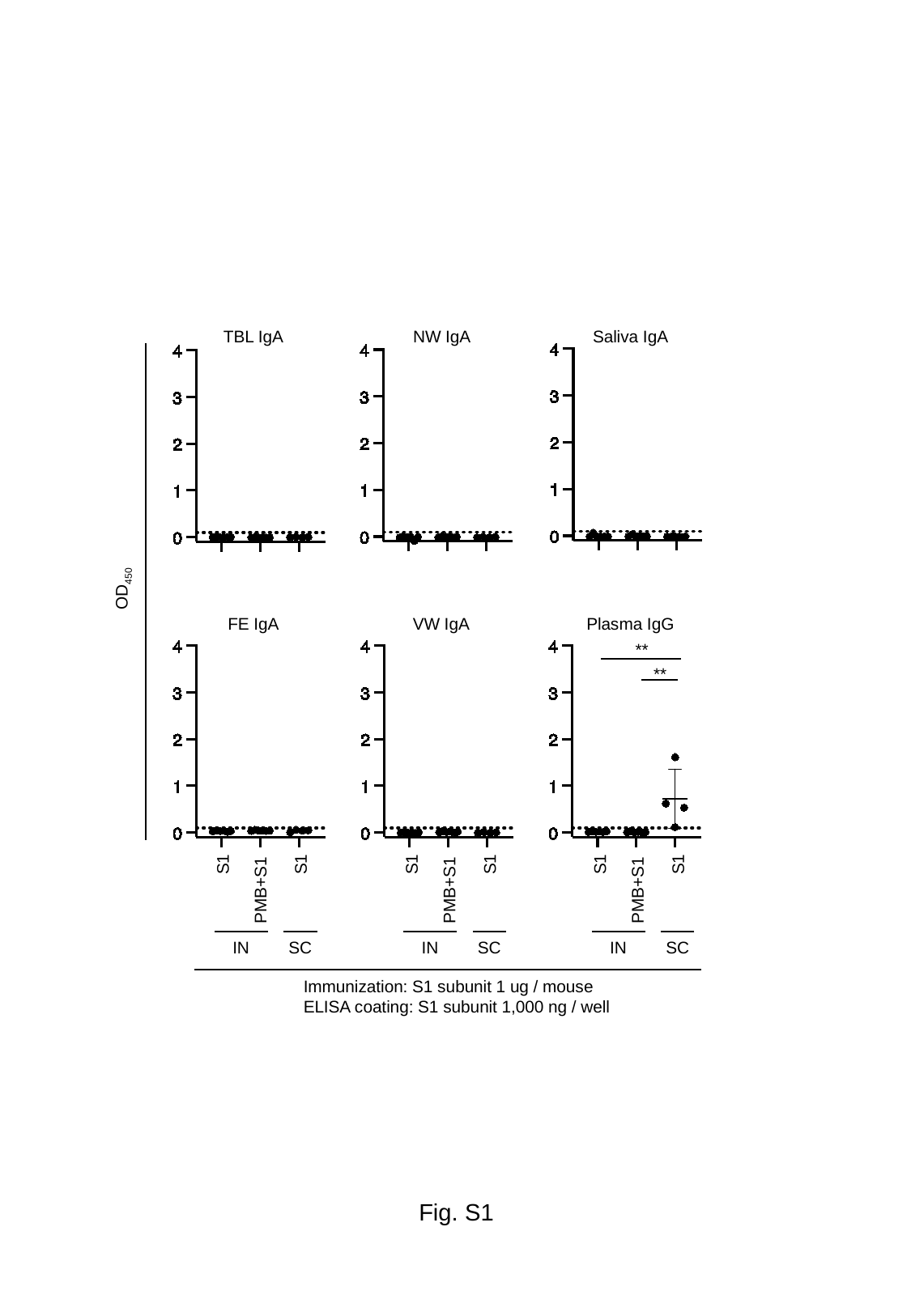

Saliva IgA
TBL IgA
NW IgA
OD450
Plasma IgG
FE IgA
VW IgA
**
**
S1
S1
S1
S1
S1
S1
PMB+S1
PMB+S1
PMB+S1
IN
SC
IN
SC
IN
SC
Immunization: S1 subunit 1 ug / mouse
ELISA coating: S1 subunit 1,000 ng / well
Fig. S1

## Slide 2
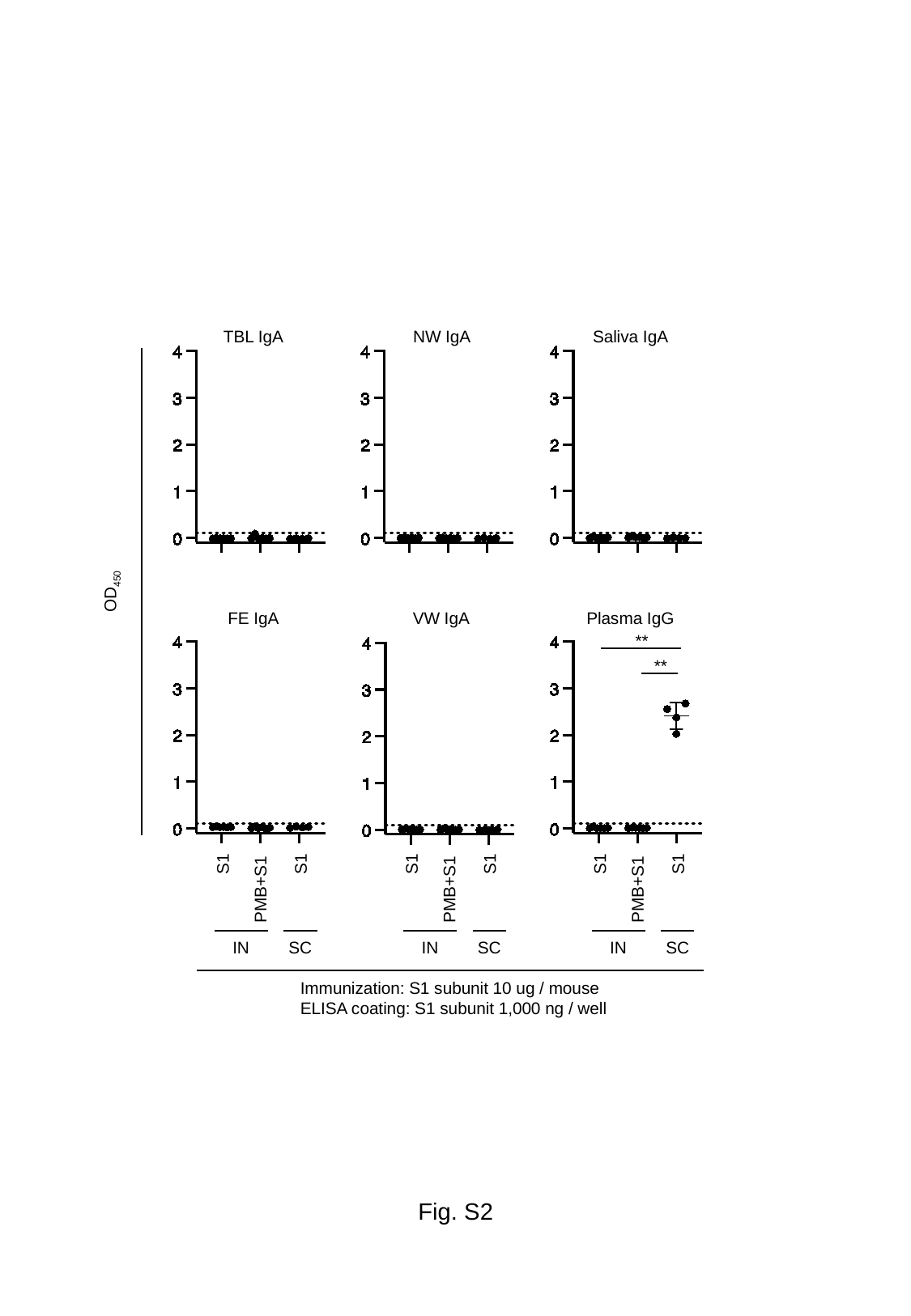

Saliva IgA
TBL IgA
NW IgA
OD450
Plasma IgG
FE IgA
VW IgA
**
**
S1
S1
S1
S1
S1
S1
PMB+S1
PMB+S1
PMB+S1
IN
SC
IN
SC
IN
SC
Immunization: S1 subunit 10 ug / mouse
ELISA coating: S1 subunit 1,000 ng / well
Fig. S2

## Slide 3
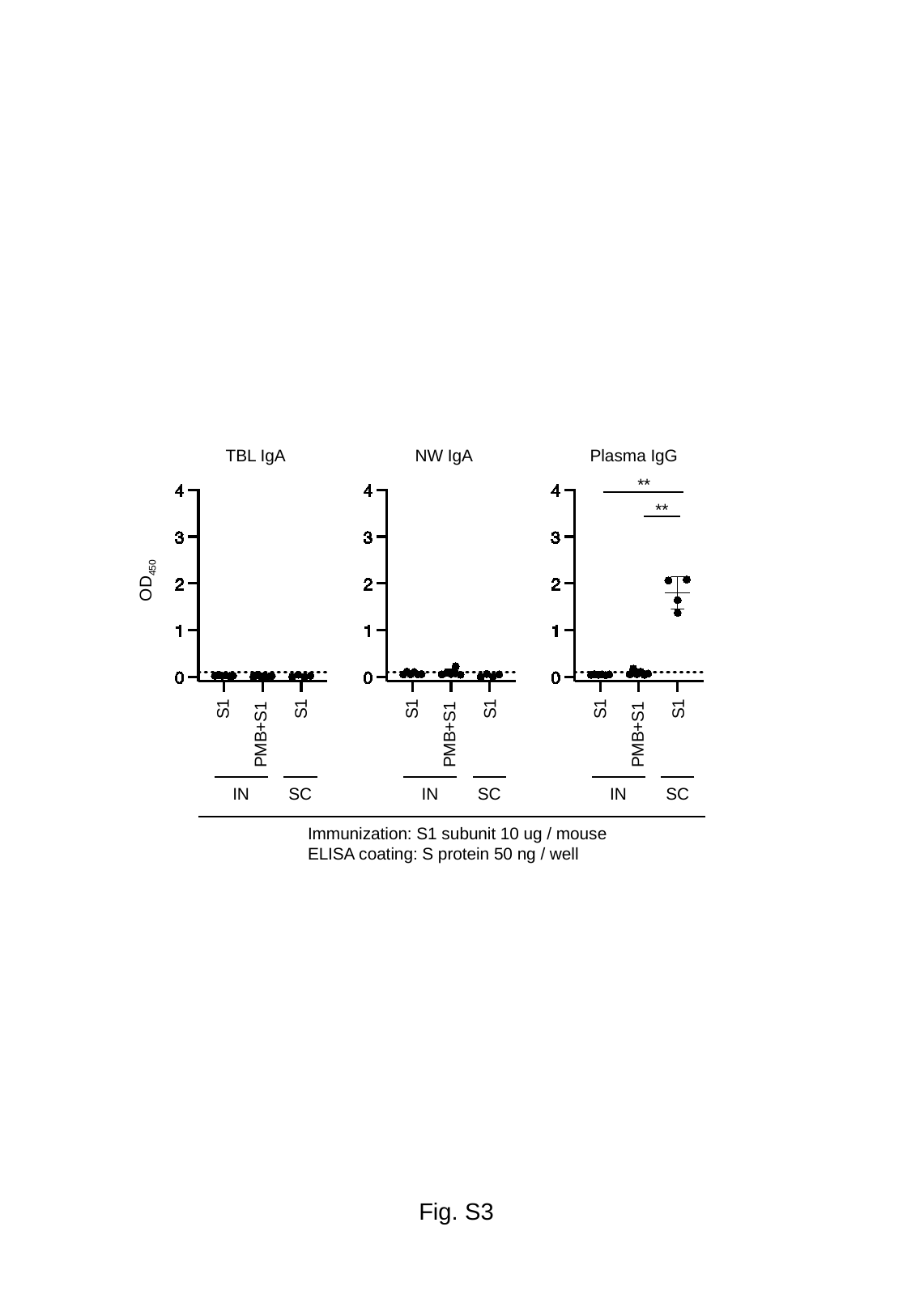

Plasma IgG
TBL IgA
NW IgA
**
**
OD450
S1
S1
S1
S1
S1
S1
PMB+S1
PMB+S1
PMB+S1
IN
SC
IN
SC
IN
SC
Immunization: S1 subunit 10 ug / mouse
ELISA coating: S protein 50 ng / well
Fig. S3

## Slide 4
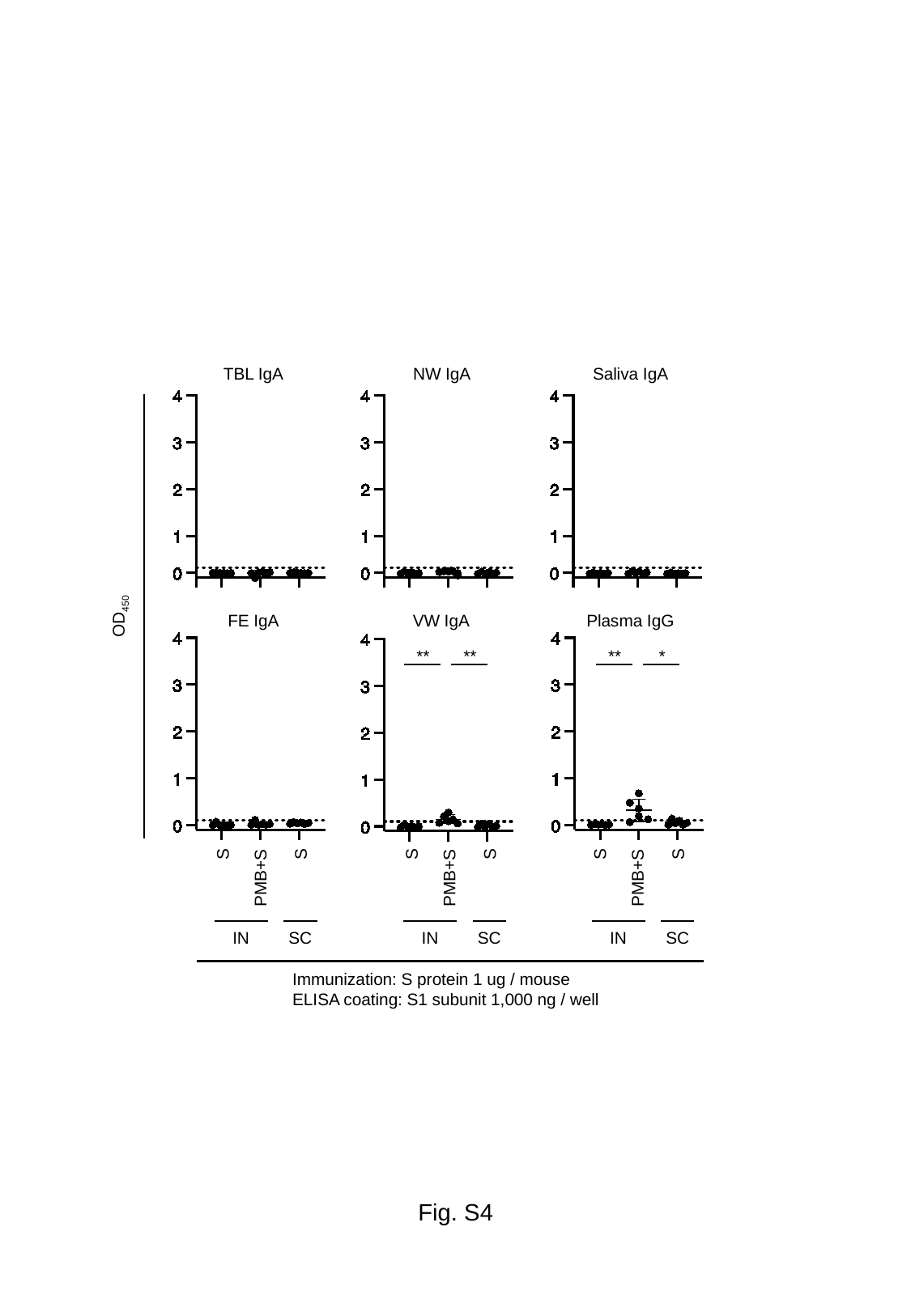

Saliva IgA
TBL IgA
NW IgA
OD450
Plasma IgG
FE IgA
VW IgA
**
*
**
**
S
S
S
S
S
S
PMB+S
PMB+S
PMB+S
IN
SC
IN
SC
IN
SC
Immunization: S protein 1 ug / mouse
ELISA coating: S1 subunit 1,000 ng / well
Fig. S4
